# Lateral closing wedge high tibial osteotomy procedure for the treatment of medial knee osteoarthritis: eleven years mean follow up analysis

**DOI:** 10.1007/s00264-025-06525-0

**Published:** 2025-04-23

**Authors:** Giacomo Dal Fabbro, Giovanni Balboni, Stefano Di Paolo, Giorgio Varchetta, Alberto Grassi, Giulio Maria Marcheggiani Muccioli, Stefano Zaffagnini

**Affiliations:** 1 IRCCS Rizzoli Orthopaedic Institute - 2nd Orthopaedic and Trauma Department, Bologna, Italy; 2 Dipartimento di Scienze Biomediche e Neuromotorie DIBINEM, Università di Bologna, Bologna, Italy

**Keywords:** High tibial osteotomy, Closing wedge, Varus knee, Osteoarthritis, Survivorship, Complications

## Abstract

**Purpose:**

To assess long term survivorship, patient reported (PROMs) and radiological outcomes, and rate of adverse events and hardware removal after lateral closing wedge high tibial osteotomy (CWHTO) for the treatment of medial knee osteoarthritis (OA) and varus malalignment.

**Methods:**

Retrospective analysis of patients who underwent isolated CWHTO for medial OA in varus knee between 2009 and 2019 at the same institution was performed. Surgical failure was defined as conversion to total knee arthroplasty (TKA) or need for osteotomy revision procedure for varus recurrence, while clinical failure was defined by a Lysholm score under 65 points. Lysholm score, Visual Analogue Scale for pain (VAS), and patients’ satisfaction with the treatment were evaluated. Radiographic parameters assessed included OA degree with the Kellgren Lawrence scale (KL), hip-knee-ankle angle (HKA), medial proximal tibial angle (MPTA), lateral distal femoral angle (LDFA), joint line convergence angle (JLCA), and posterior tibial slope (PTS). Adverse events and rate of hardware removal procedures were recorded through follow up visits and clinical records. Survival analysis was conducted through Kaplan-Meier method with surgical and clinical failure as endpoints.

**Results:**

70 knees (mean age at surgery 43.3 years) were included in the survivorship analysis at a mean follow up of 11.6 ± 3.4 years. A failure rate of 12.85% (9/70) was recorded during the follow up period, with a survivorship of 92% and 75% at ten and 15 years of follow up, respectively. Mean Lysholm score and VAS at follow up were above the PASS threshold reported in literature. The 75.7% of patients were satisfied with the treatment. Radiological follow up indicated a residual mechanical varus of 2.1°, a decrease of 0.7° of intra articular deformity (JLCA), no change in PTS nor in KL index. The adverse events rate recorded was 5.7% (4/70). In nine knees (14.7%) among the patients survived from surgical failure a subsequent hardware removal procedure was performed.

**Conclusion:**

CWHTO represents a safe procedure, which resulted in high survivorship (92% and 75% at ten and 15 years follow up, respectively), with satisfactory PROMs and radiological outcomes at long term follow up in patients affected by medial OA and varus malalignment.

**Level of evidence:**

5, Case Series.

## What are the new findings

• Lateral closing wedge high tibial osteotomy (CWHTO) yielded a survivorship from total knee replacement, osteotomy revision and low clinical outcomes of 75% at 15 years follow up, providing patient reports outcomes (PROMs) above the patient acceptable symptoms state (PASS) reported in literature.

• Despite the recurrence of a slight varus alignment, patient who underwent CWHTO showed limited osteoarthritis (OA) progression and a significant reduction of the joint line convergence angle (JLCA) compared to preoperative evaluation even at long term follow up.

• A low rate of adverse event and need for hardware removal after CWHTO was recorded. No peroneal nerve sensory or motor palsy has been reported after isolation of the external sciatic popliteal nerve from the tibialis anterior muscular compartment band.

## Introduction

High tibial osteotomy (HTO) is a proven joint preserving treatment [1] which has been shown to effectively alter ground reaction forces [2] and redistribute compressive knee forces [3] in patients affected by medial compartment osteoarthritis (OA) and varus knee. The main goal of HTO is to delay or avoid the need for joint replacement procedures, providing patients with pain relief and good joint function [1].

As the choice of the HTO surgical technique has long been related to the surgeon preference and experience [4], lateral closing wedge (CWHTO) technique has had a decrease in popularity in the last twenty years [5, 6]. The main reasons for this have been the need for two tibial bone cuts and for the fibular osteotomy, and the risk of common peroneal nerve damage, which makes this procedure technically demanding [7]. Moreover, the medial opening wedge technique (OWHTO) has shown a well-established better intraoperative control of the correction [8] and has been associated with less technically demanding conversion to total knee arthroplasty (TKA) [9], making OWHTO the current most popular procedure. Nevertheless, clear evidence of superiority in terms of survivorship from TKA of one technique over the other still lacks [10–13]. Furthermore, recent studies underlined the specific biomechanical impact of the CWHTO procedure on the proximal tibia [14], indicating that such technique would be more effective than OWHTO in addressing intra-articular deformity and in opening medial joint space [15]. All those findings showed that CWHTO procedure might still play a role in an increasingly personalised joint preserving approach for knee OA and malalignment.

The purpose of the present study was to assess survivorship, patient reported (PROMs) and radiological outcomes, and adverse events at long term follow up after CWHTO for medial knee OA and varus malalignment. The hypothesis was that CWHTO results in high survivorship from conversion to TKA and HTO revision for varus recurrence and provides good clinical and radiological outcomes at medium-long term follow up, delaying the degenerative disease of the joint with a low risk of adverse events.

## Methods

The study received Institutional Ethics Regulatory approval from Rizzoli Orthopaedic Institute Ethical Committee ( 887/2022/Oss/IOR). The study was designed as a retrospective cohort evaluation in the context of a wider trial about outcomes after osteotomy around the knee (Clinicaltrial.gov: NCT06462625). The Institutional database (II Orthopaedic Unit, IRCCS Rizzoli orthopaedic Institute, Bologna, Italy) was searched according to the following inclusion criteria: patient that underwent isolated valgus-producing lateral closing wedge high tibial osteotomy for treatment of medial knee pain, grade 2–3 OA according to Kellgren Lawrence (KL), and varus malalignment (hip-knee-ankle angle < 177°); procedures performed between 2009 and 2019, fully available clinical records, pre-operative full-length x-ray available. The following exclusion criteria were applied: osteotomy revision procedures, post-traumatic osteoarthritis or deformity, associated arthroscopic or open procedures such as meniscal, cartilage or ligamentous treatment or reconstruction, patients aged ≤ 18 y/o. Clinical and radiological follow up evaluation was performed. Patients who were not available for in person follow up were collected through phone call and stored through an online survey platform.

### Rate of failure

The surgical failure was defined as the CWHTO that have been converted to TKA or that have undergone osteotomy revision procedure for varus recurrence in function of the time. Varus recurrence was defined as the return to a preoperative varus degree. The clinical failure was defined as a reported Lysholm score under 65 points at follow up.

### PROMs

The following PROMs were investigated: Lysholm score and Visual Analogue Scale for pain (VAS). Both scores were analysed according to the patients’ acceptable symptoms scores (PASS) cut off values available in the literature [16, 17]. Furthermore, patients were asked if they were satisfied with the treatment and would perform it again.

### Radiographic assessment

The following pre and postoperative radiographic parameters were assessed on full length and lateral weight bearing radiographs: osteoarthritis degree according to Kellgren-Lawrence scale (K-L) [18], hip-knee-ankle angle (HKA), medial proximal tibial angle (MPTA), lateral distal femoral angle (LDFA) [19], knee joint line convergence angle (KJLCA) [20] and posterior tibial slope (PTS) [21].

### Adverse events and hardware removal procedures

Included patients’ clinical records and history were checked for any kind of clinical complications such as bone non-union or delayed union, deep or superficial infection, wound healing issues, common peroneal nerve (CPN) palsy. The rate of hardware removal among patients who survived from surgical failure during the considered follow up was recorded.

### Surgical technique

**Fig. 1:**
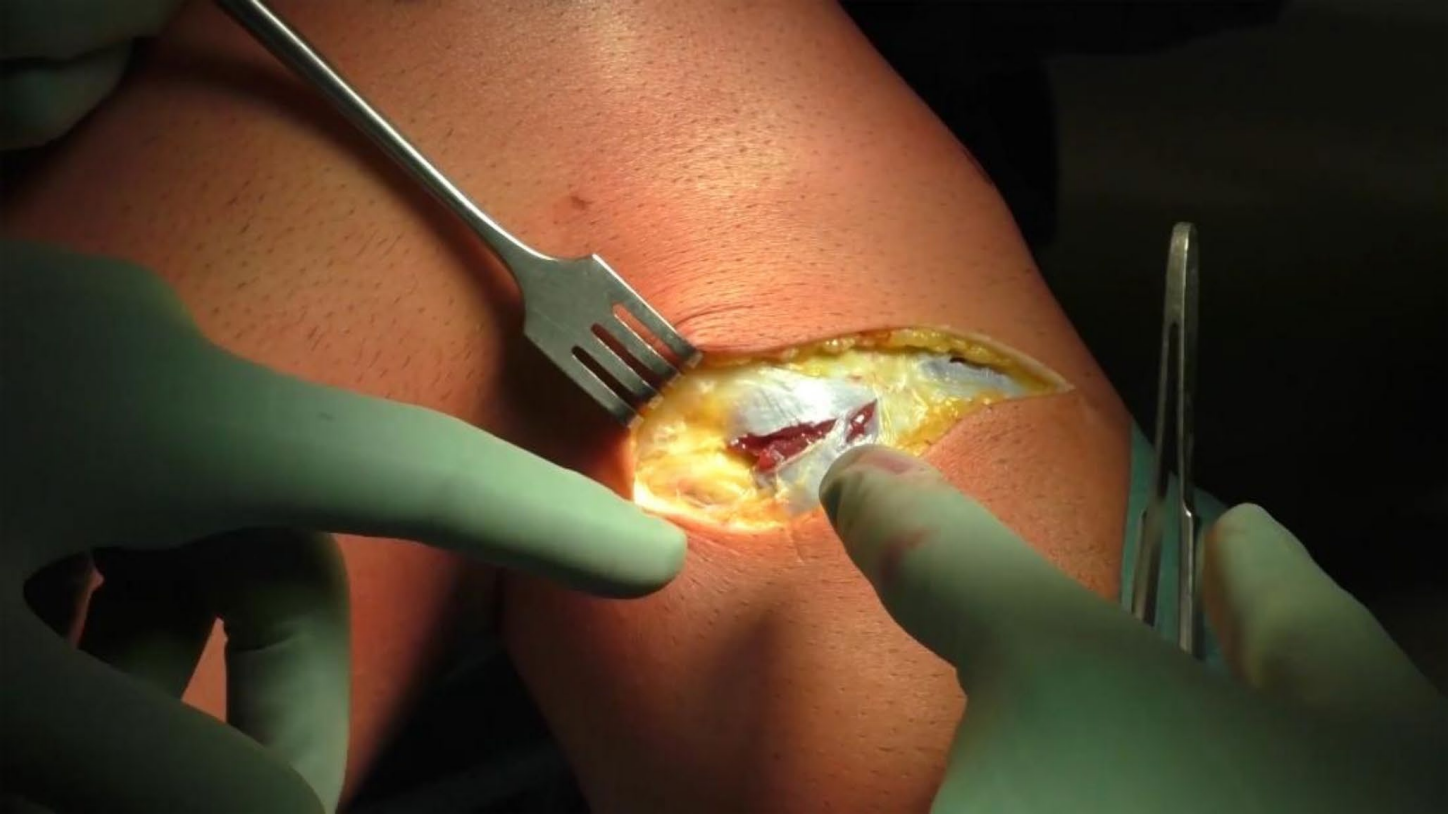
Isolation of the external sciatic popliteal nerve from the tibialis anterior muscular compartment band

All the procedures were performed by the senior author (Prof. Stefano Zaffagnini). The planned alignment correction was aimed to reach a post-operative value of HKA as near as possible to a neutral mechanical alignment (HKA 180–182°), preserving as much as possible the joint line obliquity from excessive change, considered as a post-operative mechanical medial proximal tibial angle (MPTA) higher than 95°. An oblique incision extending from the fibular head toward the tibial tubercle was performed. After blunt dissection of the subcutaneous tissue, the external sciatic popliteal nerve was isolated from the tibialis anterior muscular compartment band to avoid any possible nerve palsy caused by nerve entrapment after closing the osteotomy (Fig. 1).

**Fig. 2:**
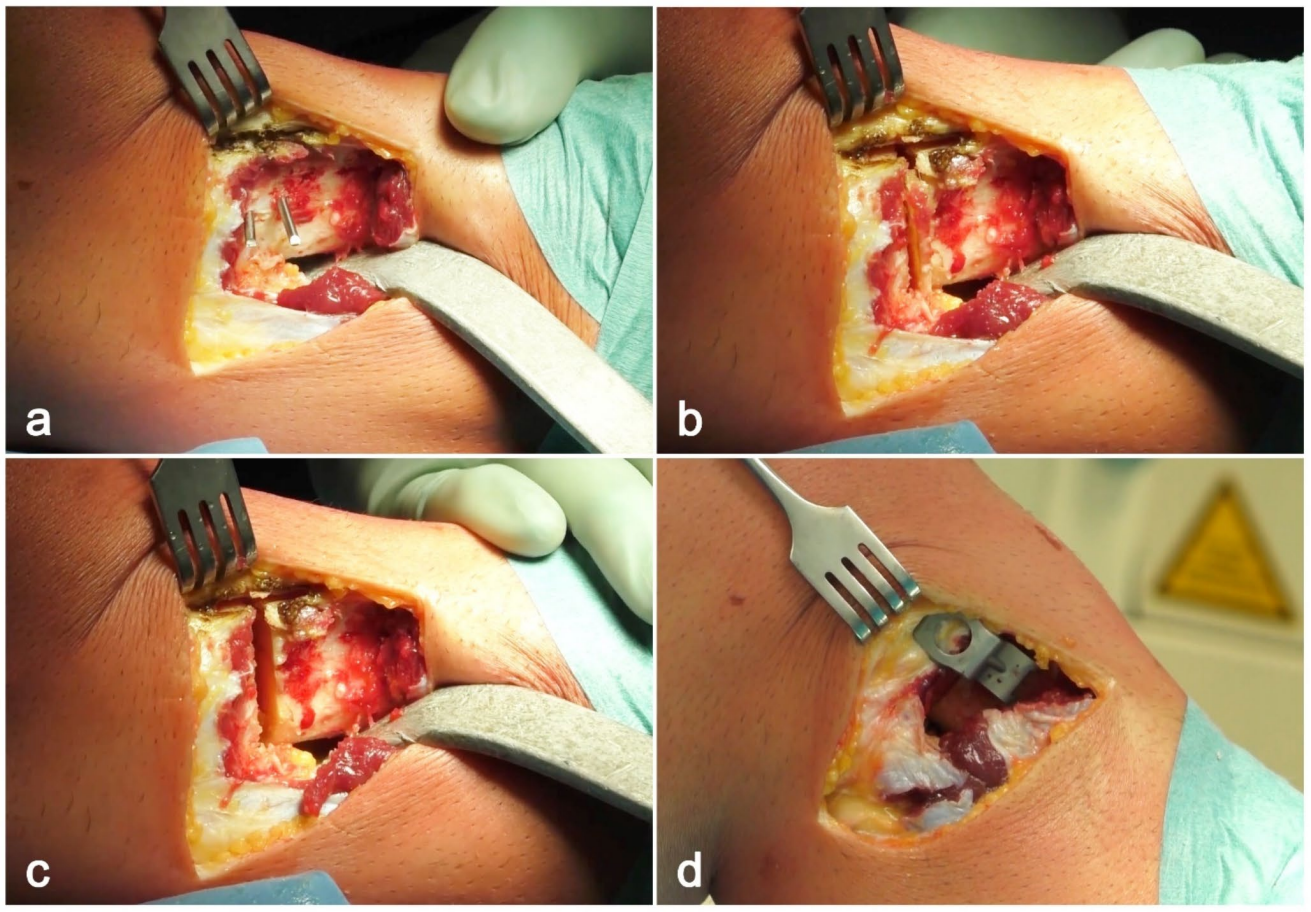
Wedge removal and osteotomy securing: (**a**) a first guide pin was inserted 2.0–2.5 cm below and parallel to the joint line, while a second pin was inserted distally to the first one, running obliquely to form an angle corresponding to the desired correction; (**b**) a sharp osteotome was used to isolate the tibial tuberosity from the bony wedge cut in the tibia to make the wedge easily removable; (**c**) The osteotomy gap after removal of the wedge; (**d**) After being closed the osteotomy was secured with a Krakow staple

**Fig. 3:**
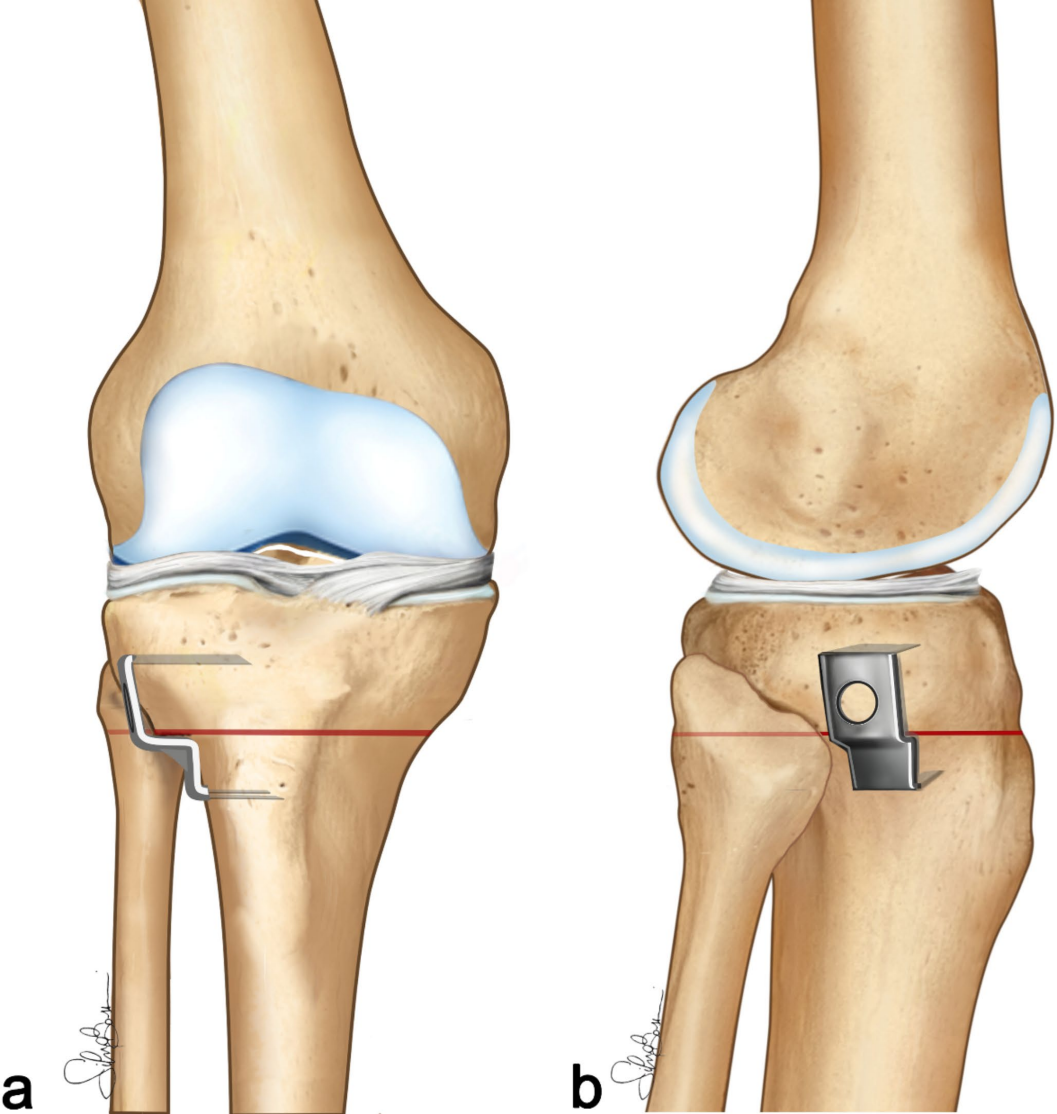
Surgical technique: (**a**) antero-posterior view; (**b**) lateral view

The fascia of the anterior tibialis muscle was opened, and the anterior tibial muscle was elevated subperiosteally from the proximal tibia. Under X-Ray guidance a first guide pin (2.5 mm in thickness) was inserted 2.0–2.5 cm below and parallel to the joint line. A second pin (2.5 mm in thickness) was inserted distally to the first one, running obliquely to form an angle corresponding to the desired correction (Fig. 2a). Using the oscillating saw, the osteotomy was performed parallel to the tibial slope in the sagittal plane. The tibia was transacted under the lower surface of wire 1 and on the upper surface of wire 2. After performing the osteotomy, a sharp osteotome was used to isolate the tibial tuberosity from the bony wedge cut in the tibia to allow an easy removal of the wedge and mobilization of the two bony fragments (Fig. 2b, c). An osteotomy of the fibula was performed at the fibular neck, with bone removal to allow for compression of the osteotomy site. Tibial reduction was achieved by applying valgus stress to the extremity with the knee at 10° of flexion. The internal fixation was accomplished with a Krakow staple (Smith & Nephew) (Figs. 2d and 3). The disruption of the proximal tibiofibular joint was avoided to prevent proximal migration and laxity in the postero-lateral structures [22].

### Rehabilitation

Following the surgery, the knee was placed in an extension brace for four weeks, removable during the day for the range of motion exercises which were allowed from the second day after surgery. Following an initial non-weight-bearing-period period of four weeks, progressive weight bearing as tolerated was allowed.

### Statistics

The statistical analysis was performed using the R-studio (4.3.2, Posit PBC, Wien, Austria). Variables were presented as mean ± standard deviation or number and percentage.

Survival analyses were performed using the Kaplan–Meier method with surgical and clinical failure as endpoints. Survival proportions at,five, ten and 15 years and mean estimated survival time with 95% confidence intervals (CI) were calculated.

Differences between single groups were inspected through student’s t-test or chi-squared test according to variable type. Differences were considered statistically significant for *p* < 0.05.

The Stuart-Maxwell test was used for comparing dependent distributions on ordinal categorical variables with more than two categories. This test is particularly beneficial when assessing whether there is a significant difference between two dependent measurements (e.g., pre-operative and post-operative) in terms of distribution across categories.

**Fig. 4:**
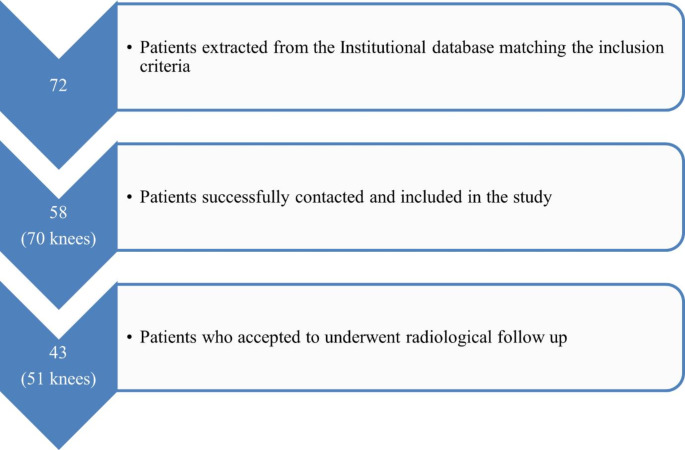
Strobe flow diagram

## Results

Of 72 patients matching the inclusion criteria, 58 (80.5%) were successfully contacted and included in the study (Fig. 4); all gave consent to participate and responded to the online surveys.

Among the 58 included patients, 12 underwent bilateral closing wedge HTO, and, overall, 70 knees were included in the analysis at a mean follow up of 11.1 ± 3.6years. Demographics and preoperative radiological data of the included patients are summarised in Table 1.

**Table 1:** Demographics

Patients (Knees)	58 (70)
Mean age (y)	43.4 ± 10.6
Sex (F/M)	8/62
Limb (Right/Left)	32/38
Mean F-U (y)	11.1 ± 3.6
BMI (Kg/m^2^)	25.3 ± 3.6
Preop HKA (°)	171.9 ± 3.2
Preop MPTA (°)	84.2 ± 4.4
Preop LDFA (°)	89.7 ± 3.0
Preop JLCA (°)	2.5 ± 2.6
Preop K-L (n)	
Grade 1 Grade 2 Grade 3 Grade 4	6 (8.5) 18 (25.7) 31 (44.2) 15 (21.4)

Preoperative radiological data are presented as mean and standard deviation. y: years; F: female; M: male; BMI: Body Mass Index; K-L: Kellgren-Lawrence; HKA: hip-knee-ankle angle; MPTA: medial proximal tibial angle; LDFA: lateral distal femoral angle; JLCA: joint line convergence angle; °: degree

### Survivorship

**Fig. 5:**
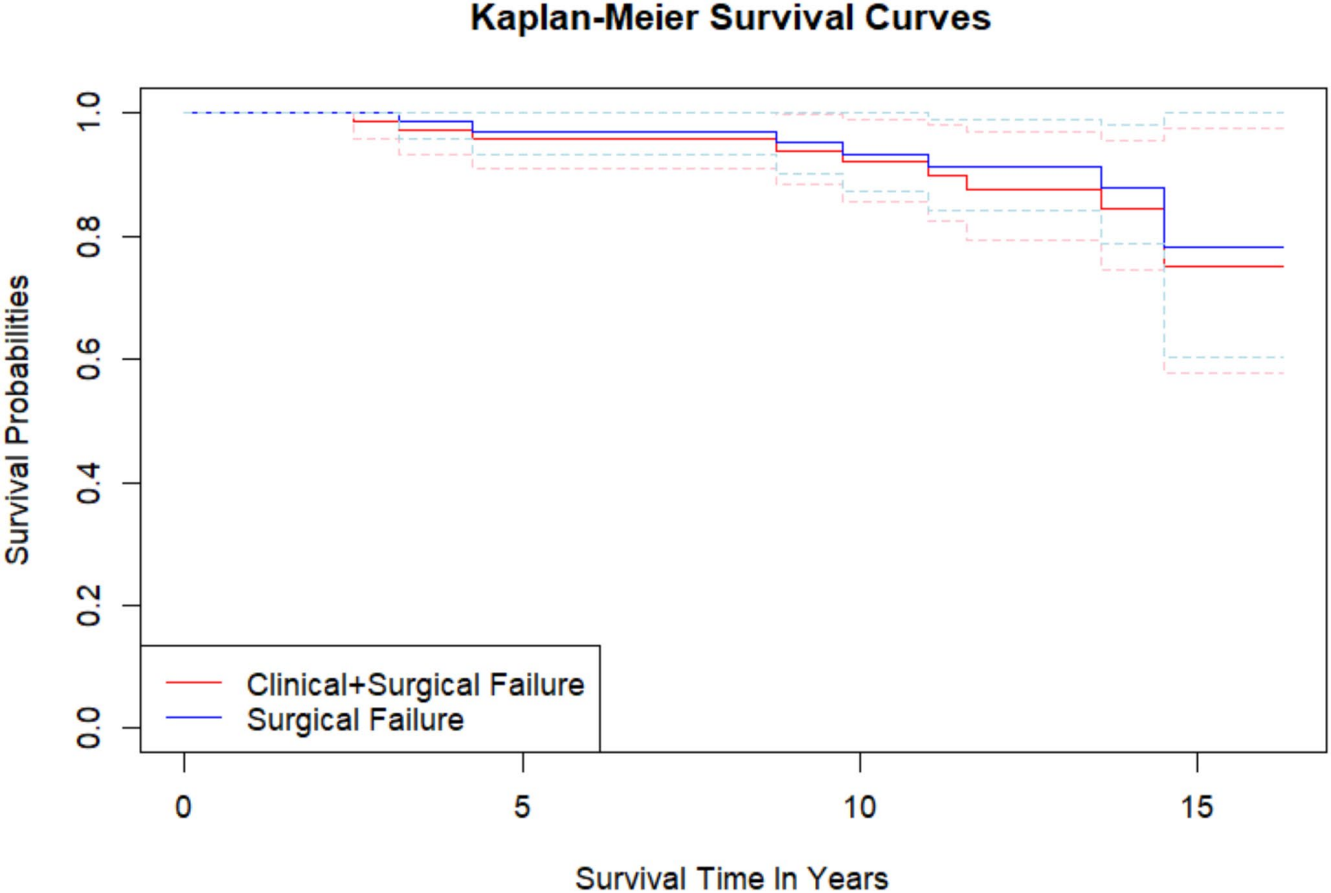
Survivorship: survivorship from surgical (blue line) and surgical + clinical (red line) failure. Dotted lines indicate the confidence intervals

Overall, during the follow up period seven knees (10.0%) underwent surgical failure (Table 2); six knees were converted to TKA, and 1 underwent HTO revision for varus recurrence. Among the survived knees, two clinical failures were reported, with a mean total failure of 12.8%. Kaplan-Meier curves are reported in Fig. 5.

**Table 2:** Survivorship rate

	*N*. of Events, %	Survival Rate, %		
		5 y	10 y	15 y
Surgical failure	7/70 (10.0%)	97.1	93.3	78.1
Surgical + clinical failure	9/70 (12.8%)	95.7	92.0	75.0

Kaplan Meier analysis: survivorship from surgical and surgical + clinical failure in population

### PROMs

The mean Lysholm score recorded at follow up was 78.9 ± 10.9, above the corresponding PASS threshold reported in literature (70.0) [17]. The mean VAS value at follow up was 3.2 ± 2.1, under the corresponding PASS threshold reported in literature [16]. 75.7% of patients reported to be satisfied with the treatment.

### Radiographic assessment

**Fig. 6:**
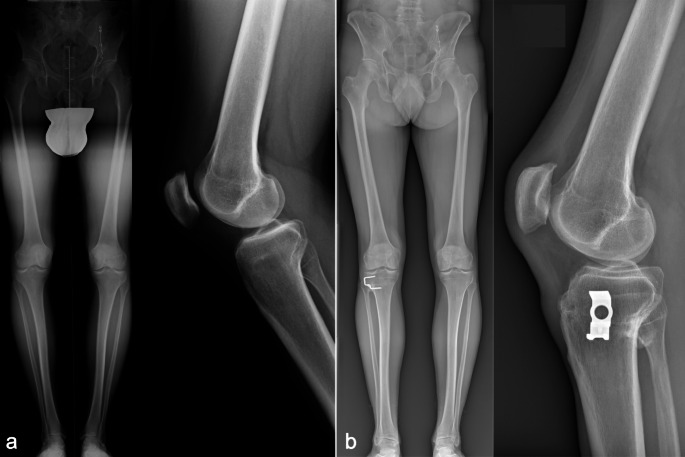
Case example: (**a**) preoperative full length weight bearing and lateral view radiograph; (**b**) full length weight bearing and lateral view radiographs at nine years of follow up

Overall, 43 patients (51 knees) were available for radiological follow up (Fig. 6). Radiological data drawn from preoperative and follow up radiographs are reported in Table 3.

**Table 3:** Radiological follow-up (51 knees)

	PRE-OP	POST-OP	DIFF [95% CI]	*P*-Value
HKA (°)	172.0 ± 3.2	177.3 ± 3.5	-5.1 [-6.3; -4.0]	< 0.001*
MPTA (°)	84.3 ± 3.3	90.3 ± 3.7	-6.4 [-7.4; -5.3]	< 0.001*
LDFA (°)	89.6 ± 3.1	90.0 ± 1.8	-0.3 [-0.9; 0.4]	0.325
JLCA (°)	2.7 ± 2.7	2.0 ± 1.6	0.7 [0.1; 1.1]	0.022*
PTS (°)	13.4 ± 4.5	13.5 ± 5.0	0.1 [-1.0; 1.1]	0.859

Data are presented as mean and standard deviation. CI = confidence intervals; HKA: hip-knee-ankle angle; MPTA: medial proximal tibial angle; LDFA: lateral distal femoral angle; JLCA: joint line convergence angle; PTS: posterior tibial slope; *: statistically significant

**Table 4:** Contingency table of KL index modification

PRE/POST	1	2	3	4	TOT
1	6				6
2	4	7	2	1	14
3		7	12	3	22
4			6	3	9
TOT	10	14	20	7	51

Contingency table according to Stuart-Maxwell test: in the columns, the pre-operative KL index grade is indicated, while the rows represent the follow-up values. Along the diagonal, the number of patients who maintained the same KL value pre-operatively and at follow-up is shown. Patients located outside the diagonal represent those who experienced a change in KL index at follow-up compared to their pre-operative values, either an increase or a decrease; KL: Kellgren Lawrence Index

The Stuart-Maxwell test was used in Table 4. The result of this test yielded a *p*-value of 0.119, which is therefore not statistically significant.

### Adverse events and hardware removal rate

Among the included patients, four (5.7%) postoperative adverse events were reported: one patient experienced a delayed bone union which was managed with prolonged non weight bearing period and serial radiological follow up. Two wounds healing condition and one superficial infection were recorded in the early postoperative phase, which were addressed with observation and antibiotic treatment, respectively. No peroneal nerve palsies have been reported. Among patients experiencing surgical failure, nine knees (14.7%) underwent hardware removal procedure during the follow up period. Removal procedures were performed in case of regional pain syndrome due to the hardware used to secure the osteotomy.

## Discussion

The main finding of the current study is that CWHTO presented a survivorship from surgical failure of 93.3% and 78.1% at ten and 15 years of follow up, respectively. Furthermore, this procedure yielded satisfactory clinical and radiological results, and was responsible for a low rate of adverse events.

Long term survivorship of CWHTO has been previously investigated, and, interestingly, the rate of survivorship from total knee arthroplasty available in the literature was lower than the results of the current study [13, 23]. A recent prospective study with long term evaluation among 100 patients affected by advanced OA reported a survival rate of 77% and 63% at ten and 15 years of follow up, respectively [23]. The higher degree of OA of the patients included in this latter study might be responsible for the lower rate of survivorship. However, in that study, the Authors performed a determinant for failure analysis, indicating a population of favourable candidates, among whom the survival rate rose up to values more similar to data reported by the present study, with 91% at ten years and 79% at 15 years of follow up. This may be due to the similar feature of the favourable candidates population and the patients included in the current study, who were mainly non obese and younger than 55 years old [23]. A recent retrospective analysis reported a rate of revision to TKA of 42.5% at nine years of mean follow up after CW procedure [13]. Such results are lower than the ones of the current study, maybe due to the older age of the patients included (57.7 versus 43.3). Another recent study pointed out similar short-term results but slightly worse long-term result in an older cohort of patients (mean age 69 years) who underwent HTO compared to a younger cohort (mean age 57 years) [24]. Therefore, the findings of the current study, associated with data available in literature, indicate that the selection of the patients might play a cardinal role in survival rate after CWHTO.

The current study pointed out satisfactory clinical outcomes at medium-long term follow up in both the assessed scores according to corresponding PASS reported in literature [16, 17]. Effectiveness of CWHTO in reducing pain and improve knee function has been well tracked in previous analysis at short, mid and long term follow up [10, 25]. According to those previous findings, in the current study a lower rate of clinical failure was detected among patients who survived from surgical failure, with only two patients reporting a Lysholm score under 65. Data about satisfaction with the treatment should be considered, as one out of four patients reported to be not fully satisfied with the treatment. In the authors opinion, these results may be due to the rehabilitation period and the time needed for the patient to benefit from the clinical improvement.

The promising clinical results in patients without surgical failure matched the data about OA progression in patients who underwent radiological follow up, with a mean degree of K-L in the medial compartment at final follow up similar to the value assessed at the baseline. These findings confirm previous data suggesting that CWHTO may have a significant role in delaying osteoarthritis and even in promoting cartilage regeneration [26, 27]. However, the real impact of HTO procedure on cartilage, the best way to assess it, and its relationship with clinical outcomes remains controversial [28], and further evidence about cartilage preservation and regeneration after high tibial osteotomy is needed.

In the present case series, the analysis of mechanical alignment showed a residual mean slight varus at long term follow up of 2.1 degree with respect to the neutral alignment considered as a target. However, this loss of correction had limited impact on the clinical outcomes. In line with these results, previous retrospective analysis reported residual varus alignment and satisfactory clinical outcomes at more than ten years of follow up after CWHTO, detecting a difference between 2° and 6° with the target correction [29, 30]. On the other hand, whilst comparative studies between closing and opening wedge procedure showed trends of higher varus recurrence after CWHTO rather than after OWHTO, they did not find statistically significant differences in alignment and correction maintenance at mid and long term follow up between those two procedures [13, 31]. The CWHTO procedure has been showed to result in greater lateral shift of the proximal tibia and medial shift of the tibial shaft than opening wedge technique [14]; furthermore, during the CWHTO technique the medial collateral ligament (MCL) site remains unaffected by the surgical procedure [32, 33]. As result of these biomechanical effect on the proximal tibia, a greater soft tissue correction has been described in CWHTO compared to OWHTO, allowing surgeons to achieve the target correction through a lower bony correction angle [14]. On the other hand, in the setting of a target correction of a neutral mechanical axis, the loss of soft tissue correction over time may be responsible for the slight recurrence of varus recorded in the literature as well as in the current study at long term follow up. A recent study [34] focused on the variation of internal and external rotation of the distal tibial bone in relation to opening wedge HTO; this was not the aim of this study but it would certainly be interesting to analyse eventual differences produced by CWHTO.

Among patients included in the current study, JLCA at long term follow up significantly decreased by 0.7° compared to preoperative evaluation. The cardinal role of reducing JLCA (ΔJLCA) to improve the outcomes has been recently pointed out in literature [35]. In a recent retrospective study, a significant improvement in clinical outcomes of patients with a ΔJLCA > 1° compared with those with a ΔJLCA ≤ 1° was reported, pointing out that medial joint opening rather than the mechanical axis deviation determined the clinical outcomes in patients who underwent HTO [15]. Moreover, the Authors of that latter study showed that CWHTO technique required a lower target correction than OWHTO in increasing the medial joint space and in reducing the JLCA. The effectiveness in reducing the JLCA may make the closing wedge technique the most suitable procedure in patients with a high pre-operative intra-articular varus deformity.

In the last twenty years the popularity of CWHTO has decreased, while OWHTO has become more commonly performed, correspondingly [5, 6]. This might have been due to technically demanding surgical aspects, as previously mentioned. A recent study [36] pointed out the benefit produced by the use of 3D printing technology in pre-operative surgical planning: this certainly might be a valid solution to help surgeon who approaches the technique for the first time. Furthermore, the risk of peroneal nerve (CPN) damage or palsy has reduced the attractiveness of the CWHTO technique [37, 38]. Nevertheless, in the current case series, a low rate of adverse event has been detected with only one serious adverse event such as delayed bone union. Others adverse events reported were two wounds healing conditions and one infection. These results are similar to data reported in literature [39]. The proximal fibular osteotomy is cardinal to obtain and maintain the correction. However, it represents a challenging surgical step due to the higher risk of CPN damage [40, 41]. Furthermore, in addition to direct trauma, compartment syndrome, stretching injury, and intra-operative ischemia of the nerve may occur during the osteotomy and its securing [42]. Remarkably, no one CPN sensory or motor palsy has been reported in the current study, suggesting that the procedure of isolation of the external sciatic popliteal nerve from the tibialis anterior muscular compartment band may play a key role in avoiding any possible nerve entrapment or damage during or after the lateral closing wedge procedure.

Unintended changes in the posterior tibial slope (PTS) represent another complication that should always been concerned in the setting of HTO [43]. However, the changes in PTS found in this study are being far from statistical and clinical significance.

Regarding the need for subsequent hardware removal procedures, the current study showed a rate of 14.7%, a value markedly lower than the rate reported in literature [44]. This finding would indicate the titanium staple as a reliable device for CW fixation. Moreover, the lower rate of hardware removal after CWHTO recorded in the current study strengthens evidence of a worthwhile cost effectiveness ratio of such technique compared to OWHTO [45].

The present study has several limitations that should be taken into account. First the low sample size, which is distributed over an interval of about ten years. The main reason for this is that only patients with pre-operative radiographs, full clinical records and surgical report available were included. Despite the low sample size, which is similar to other reported in the literature, data analysis did not affect statistical inference. Furthermore, it was not possible to contact all the patient tracked in the institutional system. However, since all the procedures included were performed by the same surgeon with the same technique and securing device, and the same post operative protocol was applied, this case series resulted in a very homogeneous sample, well representing the Authors clinical practice. The retrospective design of the study represents another significant limitation. Nevertheless, the retrospective analysis allowed the authors to perform a long term follow up analysis. Another weakness of the study was the sport and activity level of the patients before surgery. Mean age at surgery was 43 years; at this age, individuals usually do not engage in intense/professional sport activity, therefore a score based on physical activity was not considered. In any case VAS and Lysholm score takes into account pain and ability in daily life activities, giving a satisfactory overview over basic physical capabilities of the patients.

Further studies, with a prospective design and higher level of evidence are needed to confirm the findings of the present analysis.

## Conclusion

Lateral closing wedge high tibial osteotomy is a safe procedure which lead to high survivorship and good clinical and radiological outcomes. Rather than an old and technically challenging technique, it should be taken into account as a helpful tool in the setting of joint preserving treatment for medial osteoarthritis in varus knee.

## Data Availability

No datasets were generated or analysed during the current study.
